# Over-expression of the *miR-483-3p* overcomes the miR-145/TP53 pro-apoptotic loop in hepatocellular carcinoma

**DOI:** 10.18632/oncotarget.8913

**Published:** 2016-04-22

**Authors:** Laura Lupini, Felice Pepe, Manuela Ferracin, Chiara Braconi, Elisa Callegari, Sara Pagotto, Riccardo Spizzo, Barbara Zagatti, Paola Lanuti, Francesca Fornari, Reza Ghasemi, Renato Mariani-Costantini, Luigi Bolondi, Laura Gramantieri, George A. Calin, Silvia Sabbioni, Rosa Visone, Angelo Veronese, Massimo Negrini

**Affiliations:** ^1^ Department of Morphology, Surgery and Experimental Medicine, University of Ferrara, Ferrara, Italy; ^2^ Unit of General Pathology, Aging Research Center (Ce.S.I.), G. d'Annunzio University Foundation, Chieti, Italy; ^3^ Department of Experimental, Diagnostic and Specialty Medicine, University of Bologna, Bologna, Italy; ^4^ Division of Cancer Therapeutics, Institute of Cancer Research, London, UK; ^5^ Division of Experimental Oncology 2 CRO, Aviano, Italy; ^6^ Department of Medicine and Aging Science, G. d'Annunzio University, Chieti, Italy; ^7^ S.Orsola-Malpighi University Hospital, Bologna, Italy; ^8^ Department of Medical, Oral and Biotechnological Sciences, G. d'Annunzio University, Chieti, Italy; ^9^ Department of Experimental Therapeutics, MD Anderson Medical Centre, Houston, TX, USA; ^10^ Department of Life Sciences and Biotechnology, University of Ferrara, Ferrara, Italy

**Keywords:** TP53, hsa-miR-145-5p, hsa-miR-483-3p, HCC, PUMA

## Abstract

The *miR-145-5p*, which induces TP53-dependent apoptosis, is down-regulated in several tumors, including hepatocellular carcinomas (HCCs), but some HCCs show physiological expression of this miR. Here we demonstrate that in HCC cells carrying wild-type *TP53* the steady activation of the *miR-145* signaling selects clones resistant to apoptosis via up-regulation of the oncogenic *miR-483-3p*. Expression of the *miR-145-5p* and of the *miR-483-3p* correlated negatively in non-neoplastic liver (n=41; ρ=−0.342, P=0.028), but positively in HCCs (n=21; ρ=0.791, P<0.0001), which we hypothesized to be due to impaired glucose metabolism in HCCs versus normal liver. In fact, when liver cancer cells were grown in low glucose, *miR-145-5p* lowered *miR-483-3p* expression, allowing apoptosis, whereas when cells were grown in high glucose the levels of *miR-483-3p* increased, reducing the apoptotic rate. This indicates that depending on glucose availability the *miR-145-5p* has double effects on the *miR-483-3p*, either inhibitory or stimulatory. Moreover, resistance to apoptosis in clones overexpressing both *miR-145-5p* and *miR-483-3p* was abrogated by silencing the *miR-483-3p*. Our data highlight a novel mechanism of resistance to apoptosis in liver cancer cells harbouring wild type *TP53* and suggest a potential role of *miR-145-5p* and *miR-483-3p* as druggable targets in a subset of HCCs.

## INTRODUCTION

The *miR-145-5p* is deregulated in several tumors [1–6]. The tumor suppressor actions of *miR-145-5p* comprise inhibition of cell growth and metastasis [7–9], induction of apoptosis [10] and repression of pluripotency in embryonic stem cell [11]. These actions depend on the fact that *miR-145-5p* targets several genes relevant to these processes, some of which, such as MDM2, linked to the TP53 pathway [9, 11–15]. TP53 is a transcriptional activator of the *miR-145-5p*, is implicated in the miR maturation complex and is positively regulated by the *miR-145-5p* [10, 13, 15, 16]. Thus the tumor-suppressor activity of the *miR-145-5p* is linked to the TP53 mutational/expression status [10, 16, 17].

Hepatocellular carcinoma (HCC), third most common cause of cancer-related mortality worldwide [18, 19], is associated with several chromosomal, genetic and epigenetic aberrations [3, 25–35]. Mutations in the *TP53* cover only around 20% of all HCCs [20]. On the other hand lipid and glucose metabolisms are impaired in all HCCs [21–23] and HCC risk is associated with viral infections and/or metabolic disorders that promote glycolysis/lipogenesis [24].

In 50% of HCCs the *miR-145-5p* is down-regulated [25]. Here we show that in those HCCs with physiologic expression of the miRNA, the resistance to the pro-apoptotic miR-145/TP53 signaling depends on the over-expression of the *miR-483-3p*, which targets *BBC3*/PUMA [26]. Thus *miR-483-3p* could be a crucial suppressor of miR-145/TP53 signaling in the HCCs with functional TP53.

## RESULTS

### The *miR-145-5p* induces cell growth inhibition and cell death by enhancing TP53 activity in HepG2 cells

**Figure 1:**
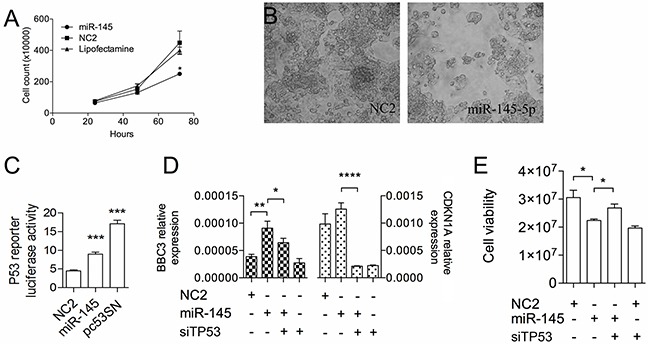
The *miR-145-5p* inhibits HepG2 cell growth by activating TP53 **A.** Growth kinetics of HepG2 cells transiently transfected with either *miR-145-5p* precursor or scramble sequence (NC2) or vehicle of transfection (Lipofectamine). **B.** Cell morphology of HepG2 cells at 72 hours after transfection with either *miR-145-5p* or NC2. **C.** TP53 dependent transcriptional activity measured by the TP53 responsive luciferase reporter vector, pP53-TA-luc, in HepG2 transfected with either *miR-145-5p* or NC2 or an expression vector carrying the human wild type TP53 cDNA (P53). Firefly luciferase activity was normalized on Renilla luciferase acitivity generated by the co-transfected vector pRL-TK. **D.** *CDKN1A* and *BBC3* expression by RT-qPCR and **E.** Luminescent cell viability assay of HepG2 cells treated (48 hours) with *miR-145-5p* alone or in combination with siRNA against *TP53* (*: p<0.05; **: p<0.01; ***: p<0.001; ****: p<0.0001).

The *miR-145-5p* has been involved in pro-apoptotic signaling through TP53-dependent mechanisms [3, 10, 15, 27–29]. Here, to confirm this mechanism in liver cancer cells, we studied the effects of the enforced expression of *miR-145-5p* in HepG2 cells, a *TP53* wild type hepatoblastoma cell line. Following cell transfection, we found that *miR-145-5p* induces a significant cell growth inhibition (p<0.05) after 72 hours (Figure 1A–1B).

In HepG2 cells we confirmed the link between the *miR-145-5p* and the TP53 pathway. Enforced expression of the *miR-145-5p* determined increased luciferase activity of the pP53-TA-luc, a TP53 responsive reporter vector (p<0001; Figure 1C) together with augmented mRNA levels of two TP53 downstream targets, *CDKN1A* and *BBC3* (Figure 1D). Moreover, silencing of *TP53* could partially rescue the effects of *miR-145-5p* on cell viability (Figure 1E)

### The *miR-483-3p* protects HepG2 cells from *miR-145-*induced cell death by targeting *BBC3*/PUMA

**Figure 2:**
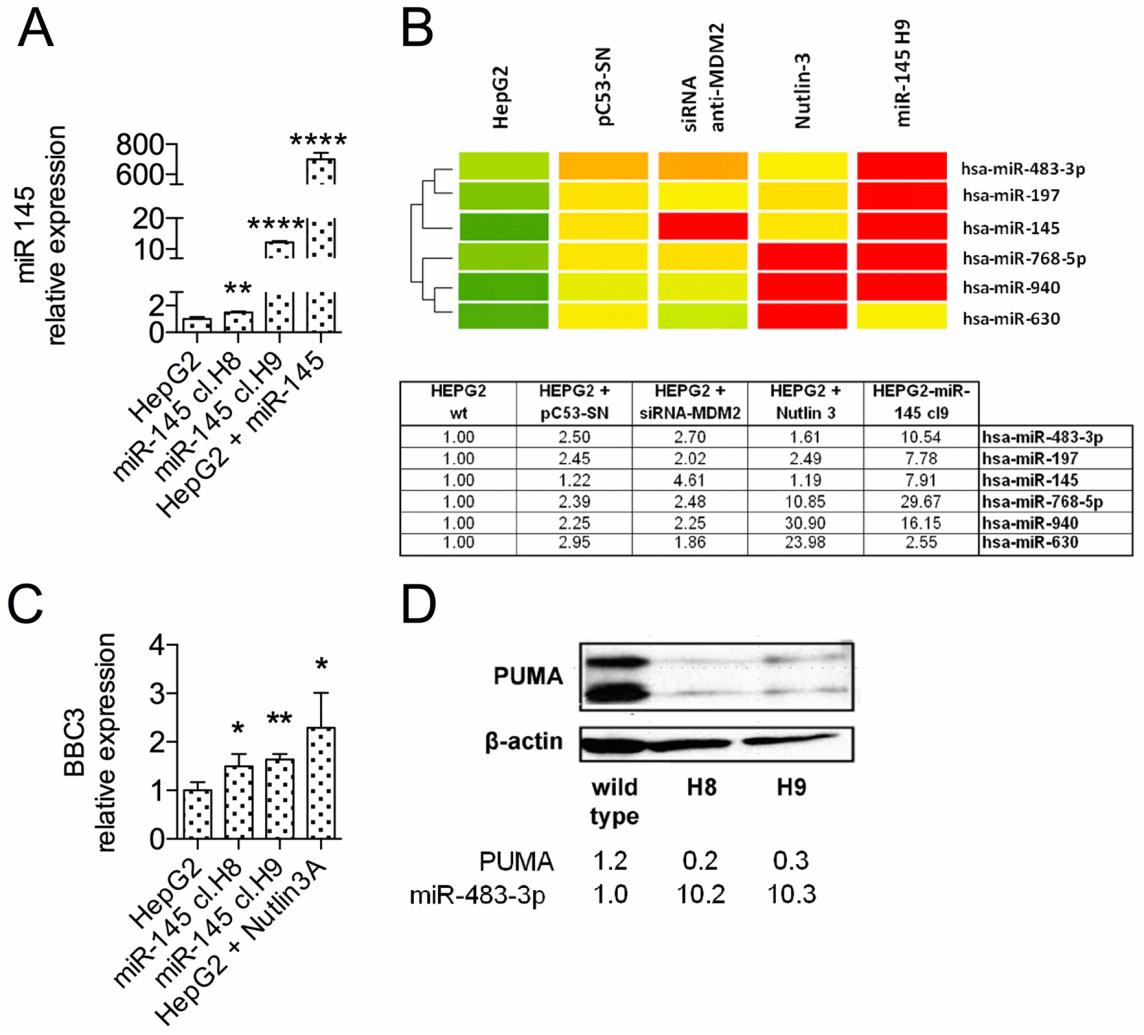
The *miR-483-3p* expression is induced in HepG2 stable clones over-expressing *miR-145-5p* **A.** The*miR-145-5p* expression was evaluated by RT-qPCR in HepG2-miR-145 stable cell clones H8 and H9, in comparison to wild type cells and to miR-145 transiently transfected cells. H8 and H9 clones exhibited a 1.5-12 fold increase of *miR-145-5p* expression, compared to wild-type cells. **B.** Heatmap representation of the miRNAs increased in *miR-145-5p* over-expressing cells assessed using microRNA microarray. Fold-change analysis revealed an increased expression of 6 microRNAs both in HepG2 cells with an activated TP53 (PC53SN, siRNA anti-MDM2 and Nutlin-3) and HepG2 cells over-expressing *miR-145* (miR-145 H9 stable clone) compared to HepG2 cells. **C.** PUMA expression was evaluated by RT-qPCR and **D.** by Western Blot in H8 and H9 cell clones in comparison to HepG2 cells. Nutlin-3A-treated-HepG2 cells were used as positive control in quantitative PCR analysis. PUMA expression levels were normalized according to β-actin expression in the same samples (*: p<0.05; **: p<0.01; ***: p<0.001; ****: p<0.0001).

To study the anti-tumor effects induced by the *miR-145-5p* in a subset of HCCs showing its physiological expression, we generated stable HepG2 cell clones carrying either the *miR-145-5p* or the control vector. Selection yielded hundreds of clones for the control vector but only 2 viable clones for the *miR-145* (H8 and H9) (Figure 2A), suggesting that these clones developed resistance to the *miR-145-5p* constitutive expression. To identify potential interplay amongst miRNAs, we performed miRNA profiling on RNA from HepG2 cells and HepG2 H9 clone. We included in the microarray analysis cells with high expression of the *miR-145-5p* determined by either exogenous expression of TP53 or MDM2 silencing or Nutlin-3a treatment (Supplementary Figure S1). The analysis revealed 6 up-regulated miRNAs, with the *miR-483-3p* in the top list (Figure 2B). In the H9 clone the *miR-483-3p* exhibited a 10-fold increased expression compared to HepG2 cells. We recently reported the oncogenic activity of *miR-483-3p* due, at least in part, to its target PUMA [26]. Therefore we evaluated protein and mRNA levels of PUMA in the H8 and H9 clones. Both clones exhibited increased levels of *BBC3* mRNA (Figure 2C), but reduced levels of PUMA protein compared to HepG2 cells (Figure 2D).

**Figure 3:**
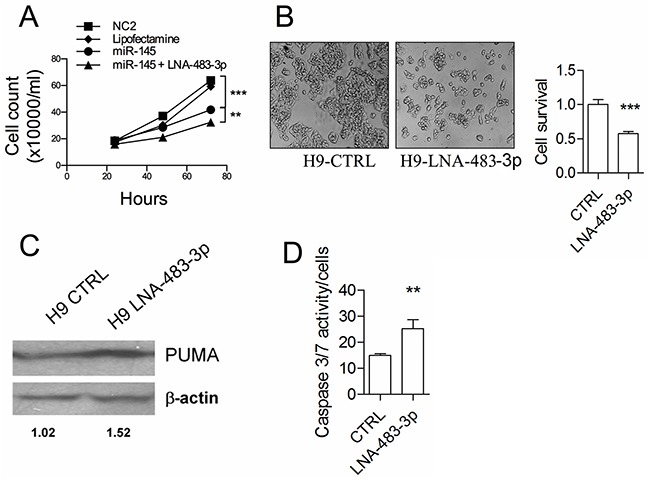
The *miR-483-3p* protects *miR-145-5p* induced cell death by targeting BBC3 **A.** Growth kinetics of HepG2 cells transiently transfected with either *miR-145-5p* precursor or and LNA-483-3p or scramble sequence (NC2) or vehicle of transfection (Lipofectamine). **B.** Cell viability by inverted microscope analysis and luminescent cell viability assay of H9 HepG2 stable clone transfected as described in A). **C.** BBC3 protein (PUMA) expression by western blot of the H8 and H9 HepG2 stable clones and HepG2 wild type cell line. Protein loaded was normalized by measuring the β-actin expression. Densitometric analysis is indicated. **D.** caspase 3/7 activity of the H9 stable clone transiently transfected with either LNA-483-3p or LNA scramble sequence (CTRL) (*: p<0.05; **: p<0.01; ***: p<0.001; ****: p<0.0001).

To assess the role of *miR-483-3p* in cell protection, we transfected HepG2 cells with *miR-145-5p* together with an anti-*miR-483-3p* oligonucleotide. The combination resulted in increased cell growth inhibition (p<0.002) (Figure 3A). We also inhibited *miR-483-3p* expression by LNA oligonucleotide in the H9 clone. This treatment reduced cell growth up to 40% at 72 hours (p<0.0001) (Figure 3B), caused an increase of PUMA expression (Figure 3C), and induced caspase 3/7 activity (Figure 3D). A key role of PUMA was confirmed by a specific si*BBC3*, which abrogated the induction of caspase activity after the inhibition of *miR-483-3p* (Supplementary Figure S2). These results support the hypothesis that the *miR-483-3p* confers resistance to TP53-dependent apoptosis in HepG2 cells over-expressing the *miR-145-5p*.

To validate these data in another liver cancer *in vitro* model we looked at the expression of the *miR-483-3p* in liver cancer cell lines harbouring either wild type (Huh-6) or mutant (SNU-449) or null (Hep3B) TP53 (Supplementary Figure S3A). We chose the HCC Hep3B cell line because of the higher expression of the *miR-483-3p* (Supplementary Figure S3A). Since they are TP53 null we reestablished wild type TP53 protein expression and functional activity by the pc53SN vector (Supplementary Figure S3B). In this model PUMA was not regulated by exogenous TP53 (Supplementary Figure S3B) but still targeted by *miR-483-3p* (Supplementary Figure S3C). AMO-483 was able to further increase the PUMA levels in presence of TP53 protein (Supplementary Figure S3C), the caspase 3/7 activity followed the same trend (Supplementary Figure S3D). The effect of the *miR-483-3p* in Hep3B cells TP53 null could be imputable to the regulation of PUMA by the TP73 protein instead of the TP53 (TP53 family: TP53, TP63 and TP73) [30–32].

To further validate the hypothesis that *miR-483-3p* protects from apoptosis in a system in which the *miR-145/*TP53 signaling acts properly, we used SNU-449 cellular model that harbour a mutant form of TP53 (Sanger Cosmic Database) and a very low expression of the *miR-483-3p* (Supplementary Figure S3A). We registered no differences between the caspase activity of cells transfected with either the *miR-483-3p* or the control and even an induction when those cells were co-transfected with the pc53SN vector (Supplementary Figure S4).

### The *miR-145-5p/miR-483-3p* circuitry is lost in HCCs

**Figure 4:**
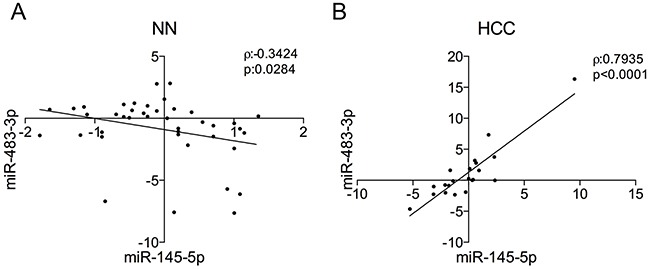
miR-145-5p/miR-483-3p correlations in non-neoplastic liver and hepatocellular carcinoma The *miR-145-5p* and *miR-483-3p* expressions obtained by microarray analysis or RT-qPCR were evaluated in **A.** non-neoplastic liver samples (NT) and **B.** hepatocellular carcinomas (HCC) respectively. Spearman correlation (ρ) and correlation p value (p) are indicated.

We searched for an association between *miR-145-5p* and *miR-483-3p* also in primary HCCs and non-neoplastic liver. We analyzed the expression of *miR-145-5p* and *miR-483-3p* on 41 RNA samples from non-neoplastic (NN) hepatic tissue, 40 cirrhotic liver samples (CL) and 1 normal liver (NL) (Supplementary Table S1); a weak but significant negative correlation between the expression of the two miRNAs was detected (ρ=−0.342, P=0.028; Figure 4A). Since enforced expression of the *miR-483-3p* abrogated the TP53/*miR-145* pro-apoptotic loop in HepG2 cells, we searched for evidence of such mechanism in primary HCCs. By analyzing RNA samples from 21 HCC, we found a strong positive correlation between the levels of the two miRNAs (ρ=0.793, P<0.0001; Figure 4B), which supports the hypothesis that high levels of the oncogenic *miR-483-3p* counterbalance high expression of the pro-apoptotic *miR-145-5p* and that in normal cells there is a negative feedback loop between *miR-145-5p* and *miR-483-3p* that is lost in tumor cells.

To strengthen our data, we evaluated the spearman correlation factors in other studies that reported miRNA profiling in HCC and NN liver (ArrayExpress: E-GEOD-30297; E-TABM-866) [33, 34]. In Pineau’ study the correlation factor is negative in both NN liver (CL, n=90; NL, n=21) and HCC (n=104), but less strong in HCC than in NN liver (CL, ρ=−0.55, P<0.0001; HCC, ρ=−0.31, P:0.0013)(Supplementary Figure S5A, S5B), whereas the data from Barry et al. confirmed the loss of the negative correlation between *miR-145-5p* and *miR-483-3p* typical of NN liver (Figure 4A; Supplementary Figure S5A), by showing a positive correlation in their set of HCC sample (n=97; ρ:-0.025, P=0.8) (Supplementary Figure S5C). *In vivo* studies were conducted by Wang et al., who performed hepatic microRNAs profiling at very early stages of hepatocarcinogenesis induced by choline-deficient and amino acid-defined diet (CDAA) in C57BL/6 mice [35]. We analyzed this dataset for the expression of the *mmu-miR-145* and mmu-miR-483* in five mice for each diet group at each time point (6, 18, 32 and 65 weeks) and we identified a negatively correlated expression of the two miRNAs in the control group (CSAA) at all time-points, that was dampened in the CDAA treated group (Supplementary Figure S6).

### TP53/*miR-145-5p* signaling rules *miR-483-3p* expression and cell death dependent on the glucose concentration of the medium

**Figure 5:**
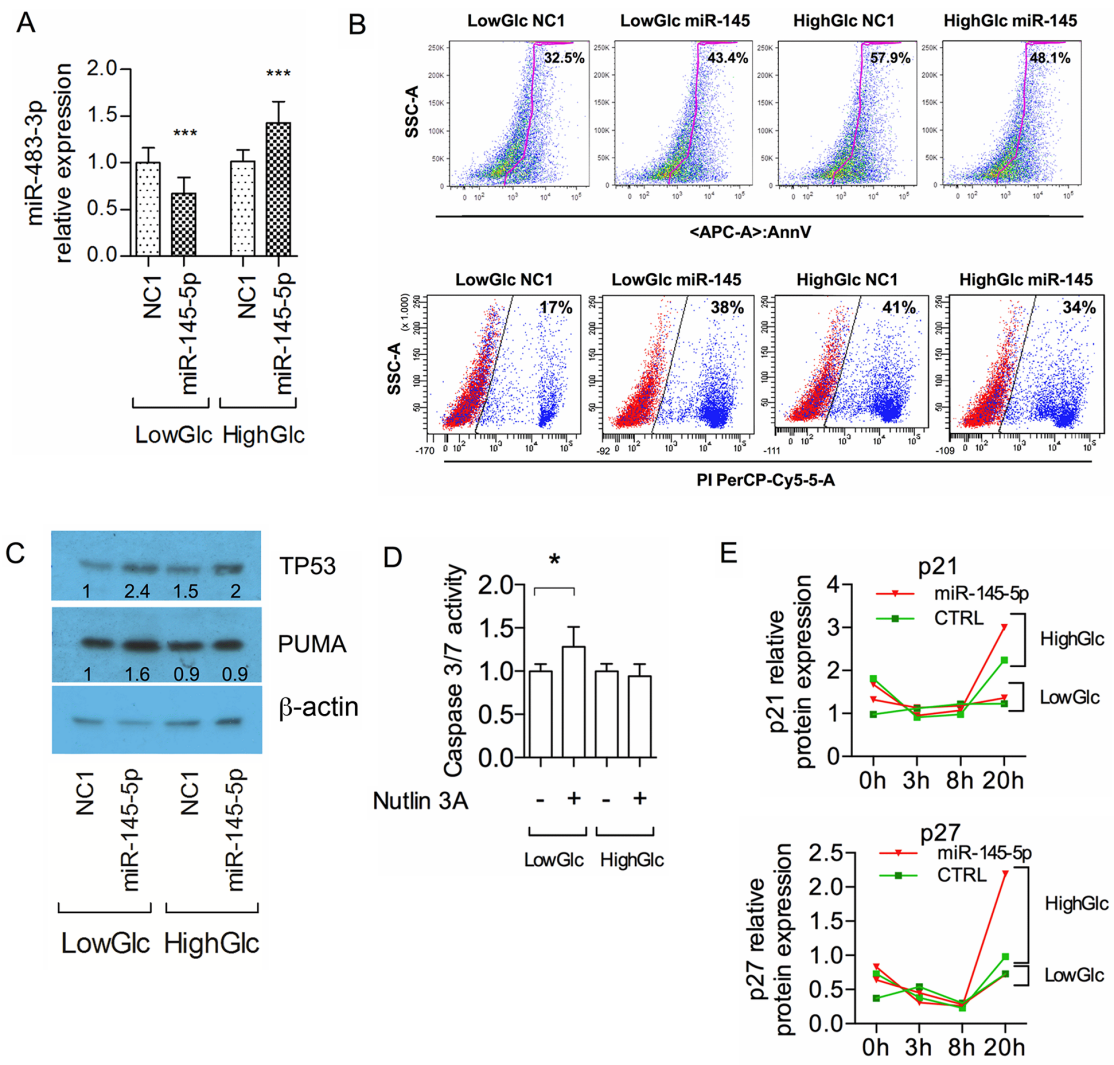
Glucose media concentrations rule the *miR-145-5p* effects on HepG2 cells **A.** *miR-483-3p* relative expression by RT-qPCR normalized on RNU44 in HepG2 cells transfected with either *miR-145-5p* or negative control miR (NC1); cells were grown in either low (LowGlc) or high (HighGlc) glucose concentration (1 g/L and 4.5 g/L respectively). **B.** Flow cytometry analysis of annexin-V (upper panel) and propidium iodide (bottom panel) staining apoptotic/dead HepG2 cells following transfection with either *miR-145-5p* or control (NC1) grown for 72h in different glucose concentrations. **C.** TP53 and PUMA/BBC3 protein relative concentration by Western blot in HepG2 cells treated as described in B). **D.** Caspase 3/7 activity in HepG2 cells transfected with *miR-145-5p* or control (NC1) in low and high glucose concentration. **E.** P21 (upper panel) and P27 (bottom panel) relative protein concentration normalized on β actin protein expression by Western blot in HepG2 cells blocked with thymidine for 17 hours and transfected with *miR-145-5p* (red triangles) or control NC1 (green squares) in low glucose and high glucose at 0, 3, 8 and 20 hours. In the graphs of RT-qPCR and Caspase 3/7 activity the data are represented by the means and standard deviations of technical and experimental replicates. Student t test was used for the statistical analysis (*: p<0.05; **: p<0.01; ***: p<0.001; ****: p<0.0001).

Next we investigated the events that could invert the correlation between the expression of *miR-483-3p* and *miR-145-5p* in HCC cells. Given that *miR-145-5p* acts as an inhibitor of cellular glucose uptake in HepG2 [36] and that *miR-483-3p* is regulated by cellular glucose availability (A.V. unpublished dataset), we investigated if glucose could modulate the effects of the *miR-145-5p* on the *miR-483-3p*. We transfected HepG2 cells with *miR-145-5p* mimic in either low (1 g/L) or high glucose (4.5 g/L) DMEM. *MiR-145-5p* induced down regulation of *miR-483-3p* under low-glucose and up-regulation under high-glucose conditions (Figure 5A). This mechanism was independent of *IGF2* expression since it showed an opposite trend of regulation when compared to the *miR-483-3p* mature expression (Supplementary Figure S7A, S7B). This suggests that glucose availability affects the regulation of the *miR-483-3p* by the *miR-145-5p.* The same results were obtained in Hep3B cells in which we restored the TP53 protein expression (Supplementary Figure S8A, S8B). Next we hypothesized that HepG2 cells cultured in high-glucose could overcome the tumor suppressive effects of *miR-145-5p* through the up-regulation of *miR-483-3p*. To verify this hypothesis we quantified the apoptotic HepG2 cells after transient transfection with *miR-145-5p* under either low or high glucose conditions. As expected, miR*-145-5p* induced apoptosis in low glucose, as shown by the increase in Annexin V-stained HepG2 cells (32.5% vs 43.4%), whereas in high-glucose the *miR-145-5p* decreased the number of apoptotic cells (57.9% vs 48.1%) (Figure 5B upper panel). We obtained similar results with Propidium Iodide (PI) staining (Figure 5B lower panel).

Since we showed that *miR-145-5p* is able to induce TP53 activity, we tested its effect on TP53 protein levels by western blot. Cells with higher expression of the *miR-145-5p* revealed increased TP53 protein levels under both low and high glucose (low: +143%; high: +33%, compared to the NC1 transfections), but PUMA, target of the *miR-483-3p*, increased only in low-glucose (Figure 5C). This is in line with the evidence that *miR-483-3p* expression is lowered by *miR-145-5p* in low-glucose and increased in high-glucose (Figure 5A). To further support these results we indirectly induced *miR-145-5p* expression by activating TP53 using Nutlin-3a, an inhibitor of the E3 ubiquitin-protein ligase MDM2, under both low and high glucose. As expected, caspase 3/7 activity was slightly increased only in low glucose (Figure 5D). Induced expression of the *miR-145-5p* after transfection and Nutlin-3a treatment and consequent regulation of *miR-483-3p* were measured by RT-qPCR (Supplementary Figure S7A, S7C, S7D).

Our data show how miR-145/TP53/PUMA signaling induces apoptosis only in HepG2 cells cultured in a low–glucose; however *miR-145-5p* exerts a growth inhibitory effect also in high glucose, as shown in Figure 1 and 3. Since PUMA contributes to autophagy [37] we hypothesized that autophagy could be activated by the *miR-145-5p/miR-483-3p* regulation in HepG2 cells. By measuring the GFP-LC3-II accumulation at the autophagosomal membranes we found that *miR-145-5p* was able to induce autophagy in HepG2 cells under both low and high glucose (Supplementary Figure S9A–S9B). Next we investigated the effect of the *miR-145-5p* on cell cycle regulation. We analyzed the protein levels of p21 and p27 after enforced expression of *miR-145-5p* under both glucose conditions. The p21 and p27 proteins were induced by *miR-145-5p* ectopic expression only in HepG2 cells cultured in high glucose (Figure 5E, Supplementary Figure S9C). Overall these data suggest that the *miR-145-5p* is able to induce apoptosis in HepG2 cells cultured in low glucose, while it only slows down the cell cycle under high glucose.

### Stable activation of TP53 through Nutlin-3a selects HepG2 clones with impaired miR-483/miR-145 ratio

**Figure 6:**
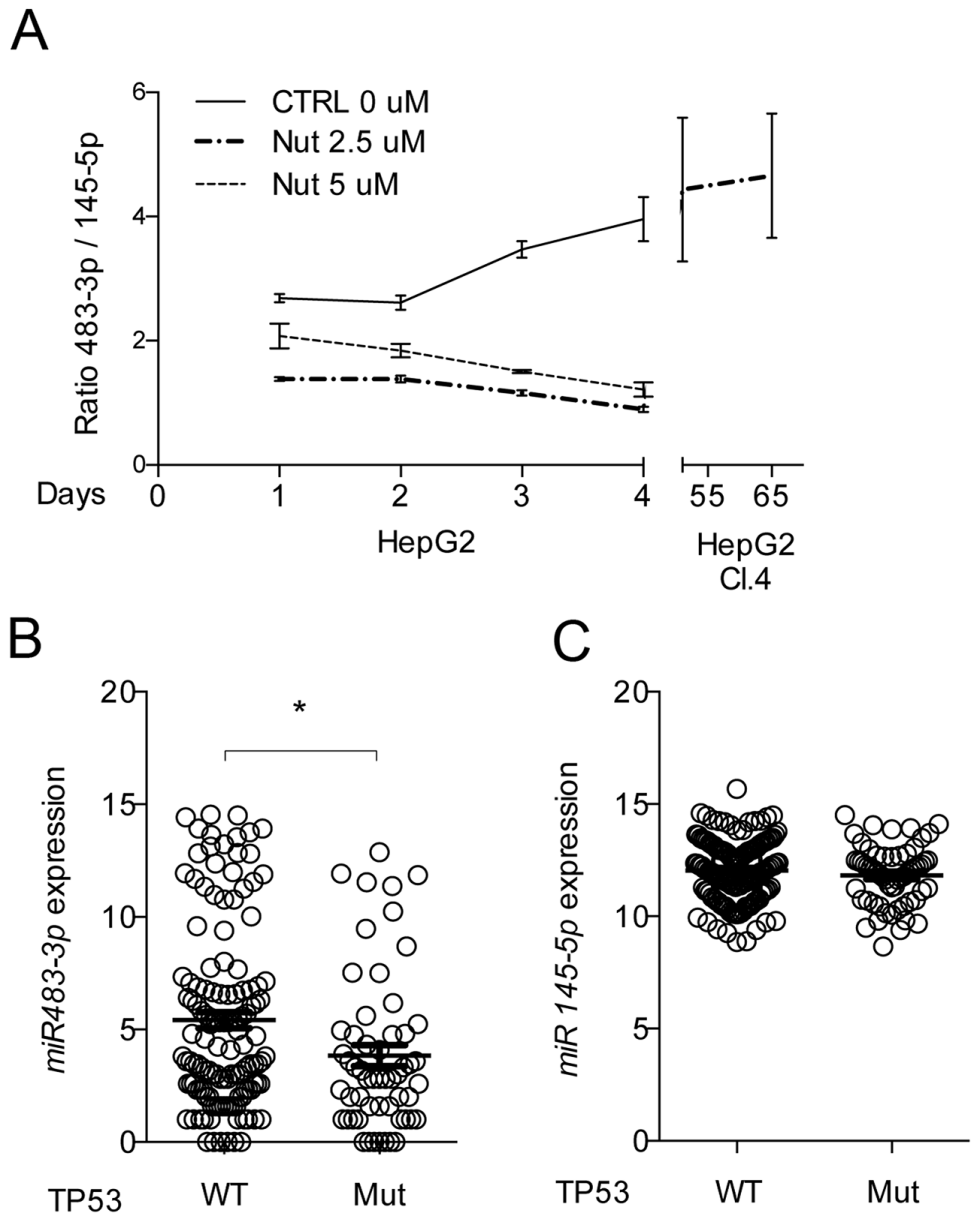
*miR-145-5p* and *miR-483-3p* expressions related to TP53 activity and mutational status **A.** Long treatment with Nutlin-3a selects HepG2 cells with high *miR-483-3p/miR-145-5p* expressions rate. *miR-483-3p/miR-145-5p* relative expressions rate of HepG2 treated with Nutlin-3a (2.5 μM and 5 μM) and vehicle (CTRL) during four days of treatment (left side). On the right side of the graph is represented the *miR-483-3p/miR-145-5p* relative expressions rate of the HepG2 stable clone 4 (HepG2 Cl.4) after 51 and 60 days of treatment with Nutlin-3a. Expression level of *miR-483-3p* **B.** and *miR-145-5p* **C.** in 199 HCC samples with (n=60) or without (n=133) mutations in *TP53* gene. The expression of *miR-483-3p* is significantly higher (p 0.01 at two-sided t-test) in TP53 WT than TP53 mutated HCC. The results shown here are based upon miRseq_mature_preprocess data generated by the TCGA Research Network: http://cancergenome.nih.gov/ and downloaded from FireBrowse portal (*: p<0.05; **: p<0.01; ***: p<0.001; ****: p<0.0001).

Finally, we used Nutlin-3a (2.5 μM) under low-glucose to constitutively activate TP53 and induce resistance of HepG2 cells to death. After 30 days we counted five clones in HepG2 cells treated with Nutlin-3a. These clones were picked up and maintained in low glucose conditions for a few days; during such period only one (clone 4, cl.4) survived. After that, we re-established Nutlin-3a until the 65^th^ day. At the same time, as a reference experiment, we treated HepG2 cells with Nutlin-3a at two concentrations (2.5 and 5 μM) for a 4 days period, sampling the RNA every day. To achieve comparability between the two experiments and determine the interplay between *miR-483-3p* and *miR-145-5p*, we took in consideration the ratio of the relative expressions of these two miRNAs (miR-483/miR-145) (Figure 6A). The HepG2 cl.4 between 50 and 65 days showed significant increases in the *miR-483-3p/miR-145-5p* ratio (Figure 6A, right side), contrary to what we observed after short term exposure to Nutlin-3a (Figure 6A, left side), that resulted a decrease of the *miR-483-3p/miR-145-5p* ratio. Our data demonstrate the ability of *miR-483-3p* to elude the miR-145/TP53 signaling, suggesting that this microRNA selects HCC cells that show a physiologic miR-145/TP53 signaling to nullify the tumor suppressive actions. To support this conclusion, we performed a meta-analysis correlating TP53 mutational status with *miR-483-3p* and *miR-145-5p* expression in 193 HCC samples from the TCGA dataset (http://firebrowse.org) (TP53 Mut: 60; TP53 Wt: 133). The *miR-145-5p* showed no difference in expression between the two groups (Figure 6C), whereas, as expected, the *miR-483-3p* was significantly up regulated in the TP53 Wt HCCs when compared to those harboring TP53-mutations (Figure 6B).

## DISCUSSION

The expression of the *miR-145-5p* is generally down-regulated in HCC and correlated to tumor grade (Edmondos grade) [3, 27], but it is not uncommon to find HCCs that show normal expression of the *miR-145-5p*. We relied on HepG2 cell clones stably expressing *miR-145-5p* to investigate the possible processes of resistance to cell death induced by TP53/*miR-145-5p* signaling in this particular HCC subset. These HepG2 clones showed up-regulation of 5 microRNAs related to TP53 activity: *miR-483-3p, miR-197, miR-768-5p, mir-940* and *miR-630*. Interestingly 4 out of these 5 deregulated miRNAs are involved in resistance to apoptosis: *miR-483-3p* is known to target *BBC3*/PUMA in HCC and CRC [26, 38]; *miR-197* is deregulated in lung cancers harbouring wild type TP53 [39]; *miR-940* and *miR-630* are up-regulated in cisplatin-resistant lung and head and neck cancers [40–42]. We focused on the most significantly up-regulated miRNA, the *miR-483-3p*. We demonstrated that the over-expression of *miR-483-3p* is an important factor to overcome the pro-apoptotic effects of miR*-145-5p*. We also identified a negative correlation between the *miR-145-5p* and the *miR-483-3p* in non-neoplastic liver that became less evident, lost or positive in HCCs. This suggests the presence of a regulative loop between these two microRNAs: in physiological conditions miR-145/TP53 signaling results in the inhibition of *miR-483-3p* (negative correlation), whereas in neoplastic conditions *miR-145-5p* selects cells that show higher expression of *miR-483-3p* (positive correlation). The expression and activity of the *miR-145-5p* is associated to TP53 [10, 15, 16] whereas *miR-483-3p* is correlated to β–catenin nuclear activity [38]. Since mutations in TP53 and β–catenin are mutually exclusive in HCC [20], we speculate that the HCCs that show TP53 mutations, and consequent down regulation of PUMA, p21 and other TP53 targets, do not need the up-regulation of *miR-483-3p* to reduce the apoptotic rate. On the contrary, as confirmed by our data, the HCCs with wild-type TP53 select cells with higher expression of the *miR-483-3p*. We found that, on the basis of glucose availability, the *miR-145-5p* represses (low glucose) or induces (high glucose) the expression of the *miR-483-3p*. We propose that increased glucose uptake in the progression from pre-neoplastic to neoplastic liver cells and the metabolic shift from aerobic to anaerobic metabolism (Warburg's effect), typical of cancer [43, 44], affect the physiologically negative regulation of *miR-483-3p* and the *miR-145*/TP53 axis, dampening the anti-tumoral effect of this pathway and facilitating tumor development. Thus, to exclude resistance via *miR-483-3p* upregulation, the possible therapeutic development of *miR-145-5p* mimics should comprise strategies directed also to the concurrent inhibition of the *miR-483-3p*.

## MATERIALS AND METHODS

### Cell lines and in vitro assays

Hepatoblastoma (HepG2, Huh-6) and hepatocarcinoma (Hep3B, SNU-449) cell lines are from ATCC (UK). HepG2 cells harbour a wild type TP53 gene, while Hep3B are null for the TP53 gene (www-p53.iarc.fr). They were cultured in IMEM/DMEM supplemented with 10% fetal calf serum. For *in vitro* cell growth assays, cells were seeded in 24-well plates at a density of 70,000cells/well. The transfection was performed by Lipofectamine2000 protocol (Invitrogen, CA, USA) with a final concentration of 100 nM for either *miR-145* precursor or negative control molecule (Ambion negative control #2). Four replicates of each condition were counted at each time point (0, 24, 48 and 72 hours). Time 0 was the time of transfection. siRNA-PUMA (Dharmacon) and siRNA-p53 (Dharmacon) were transfected at final concentration of 100 nM. To test the effect of Nutlin-3a on *miR-145* expression, we treated cells for 48 hours at a final concentration of 5 μM. The number of cells was counted using hemocytometer chamber. Cell viability was analyzed by using CellTiter- GloTM Luminescent Cell Viability Assay (Promega, WI, USA), and, apoptosis by using Caspase- Glo™ 3/7 Assay (Promega, WI, USA)

### MicroRNA precursor molecules and siRNAs

Synthetic microRNA precursor molecules and negative controls (Ambion negative control #2) were purchased form Ambion (Austin, TX). The synthetic oligos were dissolved in nuclease free water to a stock concentration of 50μM. Small interfering RNA (siRNA) against TP53 and PUMA were purchased from Dharmacon (Thermo Scientific, MA, USA). Anti-miR-483-3p LNA oligonucleotide was purchased from Exiqon (Vedbaek, Denmark).

### TP53 expression and reporter vectors

The reporter vector pp53-TA-Luc (Clontech Carlsbad, CA, USA) was used to quantify TP53 transcriptional activity. The vector contains a TP53 responsive element located upstream the TATA box from the herpes simplex virus timidine kinase promoter (pTA). Downstream of pTA is the firefly luciferase reporter gene (Luc). The pRL-TK vector contains the herpes simplex virus thymidine kinase promoter to provide low to moderate levels of *Renilla* luciferase expression in co-transfected mammalian cells. The vector was used as an internal control reporter in combination with pp53-TA-Luc reporter vector. Luciferase activity was measured using a dual luciferase kit (Promega, WI, USA) and quantified at a luminometer (Turner, Biosystems, Sunnyvale, CA, USA). Each condition was assayed in four replicates in two independent experiments. The TP53 mammalian expression vectors pc53SN, which carries a human wild type TP53 cDNA were a kind gift of Dr. Arnold Levine and were previously described [45].

### RNA isolation, retrotranscription and quantitative PCR

The RNA purification by Trizol was performed according to manufaturer's indications (Invitrogen, Carlsband, CA, USA). For mature microRNA quantification we performed a Taqman Real time PCR, using *miR-145-5p, miR-483-3p, RNU6B* and *RNU44* probes (Applied Biosystems, Foster City, CA). 25 ng of total RNA was retrotranscribed using the specific stemloop primers. For *BBC3*/PUMA, *CDKN1A*/p21 and *TP53* and quantification we used Taqman assays from Applied Biosystems (Foster City, CA). *IGF2* expression were quantified by using UPL technology (Roche) (UPL: #40; U40_IGF2_F: acaccctccagttcgtctgt; U40_IGF2_R: gaaacagcactcctcaacga). First, 0.5-1 ug of total RNA was retrotranscribed by SuperScript II (Invitrogen, Carlsband, CA, USA) with random hexamers; then, PCR amplification was performed using Taqman primers and probes as indicated by manufacturer (Applied Biosystems, Foster City, CA). microRNAs expressions were normalized to *RNU6B* or *RNU44* expressions, whereas mRNAs were normalized on *ACTB* expression. The delta Ct method was used to calculate the relative abundance.

### Western blotting and antibodies

Cells were collected by trypsin-EDTA and dissolved in NP40 lysis buffer (0.5% NP40, 250mM NaCl, 50mM Hepes, 5mM EDTA and 0.5mM EGTA) freshly supplemented with Complete inhibitor (Roche, Mannheim, Germany) and phosphatase inhibitor cocktail 1&2 (Roche, Mannheim, Germany). The following antibodies were used for the detection of p53 (polyclonal p53 Antibody #9282 Cell Signaling, Danvers, MA, 1:1000), CDKN1A/p21 (clone CP74, P1484 Sigma-Aldrich, St. Louis, MO, 1:200), PUMA (polyclonal PUMA Antibody #4976 Cell Signaling, Danvers, MA, 1:1000) and β-actin (clone AC-40, A4700 Sigma Aldrich, St. Louis, MO, 1:1000).

### Human microRNA microarray detection and data analysis

MiRNA expression was investigated using the Agilent Human miRNA microarray v.2 (#G4470B, Agilent Technologies). This microarray consists of 60-mer DNA probes synthesized in situ and contains 15,000 features which represent 723 human microRNAs, sourced from the Sanger miRBASE database (Release 10.1). One-hundred ngs of total RNA were employed in each experiment. RNA labeling and hybridization were performed in accordance to manufacturer's indications. Agilent scanner and the Feature Extraction 10.5 software (Agilent Technologies) were used to obtain the microarray rawdata. Microarray results were analyzed by using the GeneSpring GX 10 software (Agilent Technologies). Data transformation was applied to set all the negative raw values at 1.0, followed by a Quantile and on-gene median normalization. Fold-change analysis was used to identify the microRNAs activated both by TP53 (pc53SN, siRNA anti-MDM2 and Nutlin-3a) and *miR-145-5p* (miR-145 H9 stable clone).

### Statistical analysis

The Student's t test was used to compare average values between groups of samples (such as miRNA expression data, number of proliferating cells, etc). All reported p-values were calculated assuming groups with unequal variance.

## SUPPLEMENTARY FIGURES AND TABLES


