# Supplementary material for: Over-expression of the miR-483-3p overcomes the miR-145/TP53 pro-apoptotic loop in hepatocellular carcinoma
Source: Oncotarget. 2016 Apr 22;7(21):31361–71. doi: 10.18632/oncotarget.8913 (PMC5058762; doi:10.18632/oncotarget.8913)
Supplement: Supplementary file 1 [file oncotarget-07-31361-s001.pdf]

SUPPLEMENTARY FIGURES AND TABLE

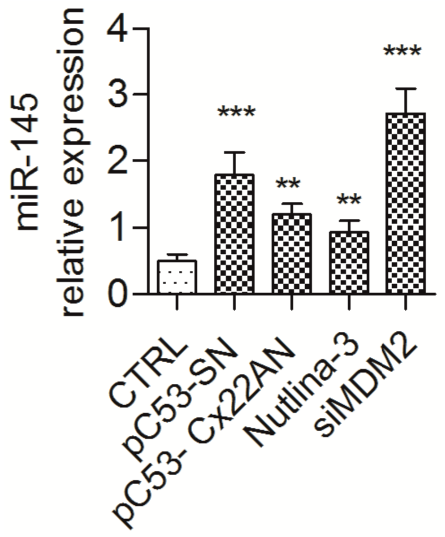

Supplementary Figure S1: TP53 induced *miR-145-5p* expression. *MiR-145-5p* levels were measured by quantitative real time PCR in HepG2 cells treated with plasmids (PC53-SN and PC53-Cx22AN), a drug (Nutlin-3a) or siRNA (siMDM2) able to induce p53 activation in cells. In all conditions miR-145 expression significantly increased compared to not treated cells (CTRL).

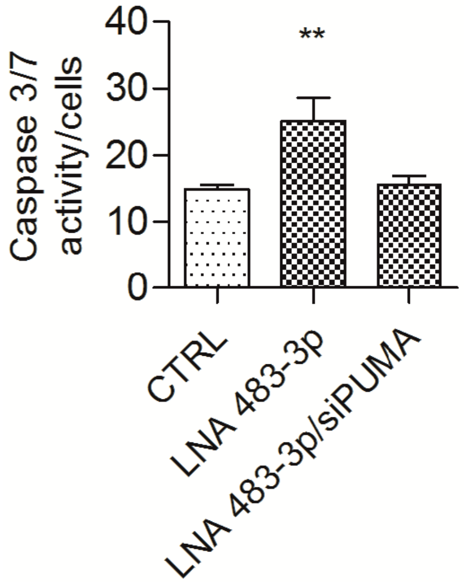

Supplementary Figure S2: LNA anti-miR-483 triggers cell death in *miR-145-5p* stable cell clone through PUMA activation. Caspase 3/7 activity was evaluated in H9 miR-145 stable cell clone after 72h from transfection with an anti-miR-483-3p LNA oligonucleotide alone or in combination with a siRNA against *BBC3* mRNA, using Caspase-Glo® 3/7 Assay kit (Promega). Anti-miR-483-3p LNA was responsible for an increase in caspase activity, that appeared significantly reduced where cotransfected with anti-PUMA siRNA.

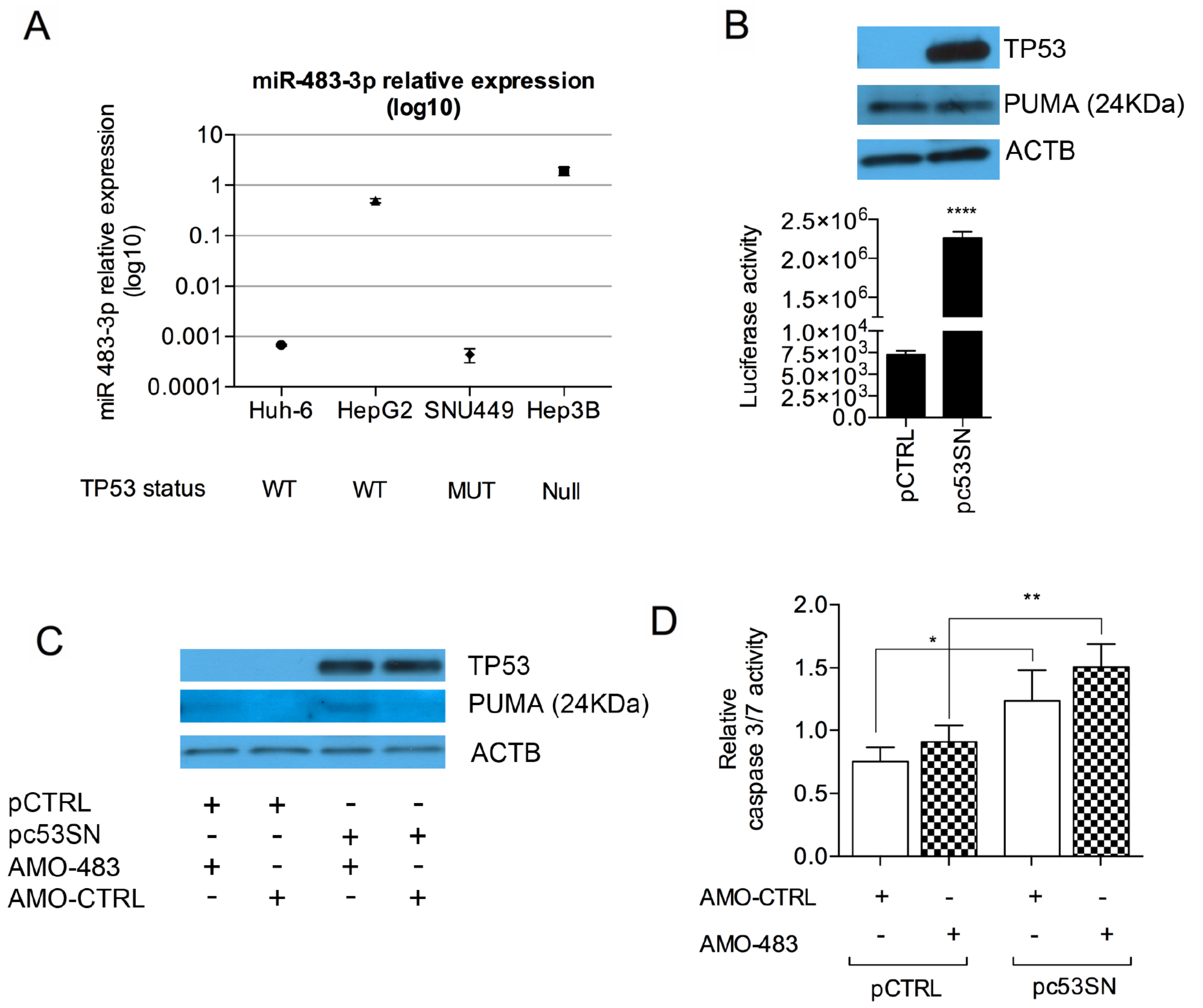

Supplementary Figure S3: Evaluation of *miR-145-5p*/TP53/*miR-483-3p* signalling of Hep3B cells. A. *miR-483-3p* relative expression by RT-qPCR in four liver cancer cell lines with different status of TP53; wild type (WT) in Huh-6 and HepG2 cells, mutated (MUT) in SNU449 and homozygously deleted (Null) in Hep3B cells. **B.** Hep3B cells transfected with the Empty expressing vector (pCTRL) or the TP53 wild type expressing vector (pC53SN) were analysed by western blot to measure the PUMA and TP53 protein levels. β-actin (ACTB) were used to evaluate the total protein loading (upper panel B). The lower panel B shows the ability to activate the transcription of the luciferase gene of the reporter vector pp53-TA-Luc containing the TP53 responsive element by exogenous expression of TP53. **C.** Western blot analysis of TP53, PUMA and β-actin (ACTB) in Hep3B cells. Cells were transfected with either the empty expressing vector (pCTRL) or the TP53 wild type expressing vector (pC53SN), the anti-miR control (AMO-CTRL) or the anti-miR-483-3p (AMO-483). **D.** Caspase 3/7 activity in Hep3B cells transfected with the empty expressing vector (pCTRL) or the TP53 wild type expressing vector (pC53SN) and the anti-miR control (AMO-CTRL) or the anti-miR-483-3p (AMO-483).

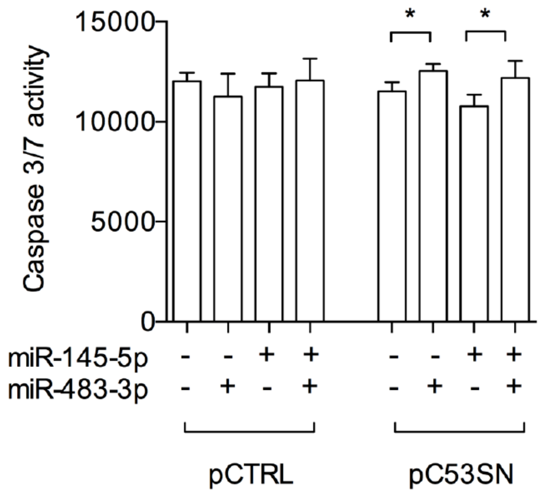

Supplementary Figure S4: Caspase 3/7 activity in SNU449 cells transfected with *miR-145-5p (+), miR-483-3p* (+) or control (NC1, -), the empty expressing vector (pCTRL) or the TP53 wild type expressing vector (pC53SN).

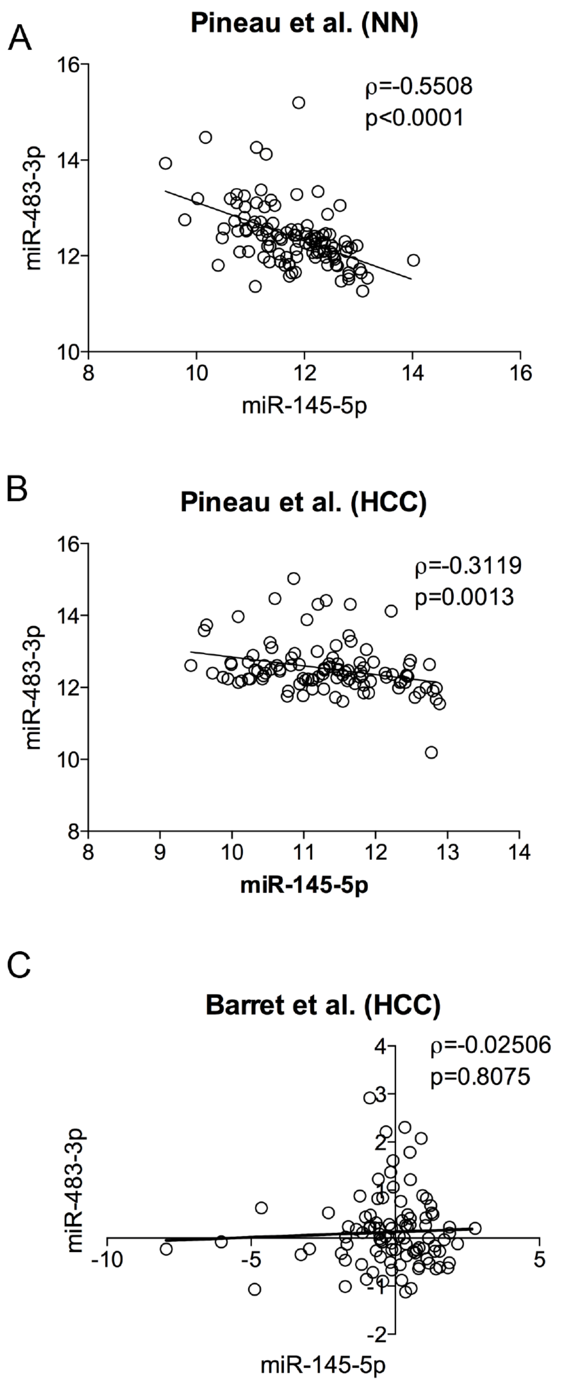

Supplementary Figure S5: Correlation between *miR-145-5p* and *miR-483-3p* expression in clinical samples of HCC or healthy liver. microRNA expression data were obtained from Array Express, from two different data set: E-TABM-866 **A, B.** (MicroRNA profiling of human hepatocellular carcinoma samples shows miR-221 overexpression contributes to liver tumorigenesis) and E-GEOD-30297 **C.** (Micro RNA expression from human hepatocellular carcinoma (HCC) specimens from patients undergoing liver transplantation). The inverse correlation between *miR-145-5p* and *miR-483-3p* in non neoplastic livers (NN) (A) was lost in hepatocellular carcinoma samples (HCC) (B–C).

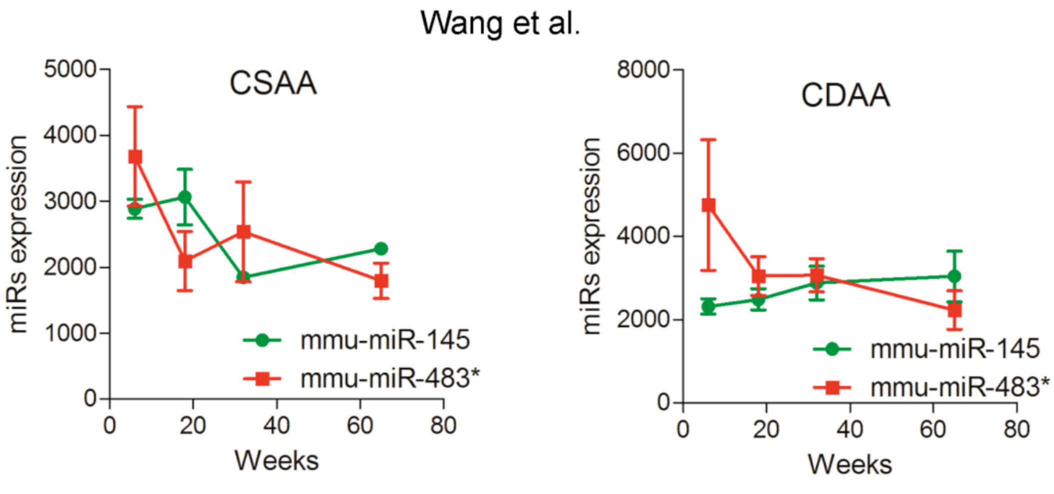

Supplementary Figure S6: mmu-miR-145 and mmu-miR-483* expressions in C57BL/6 male mice (6 weeks old) fed with CSAA (choline sufficient, amino acid-defined diet) or CDAA (Lombardi's choline-deficient, low methionine and amino acide-defined diet) diet. As described by Wang et al., five mice were used in each diet group for each time point (6, 18, 32, 65 weeks).

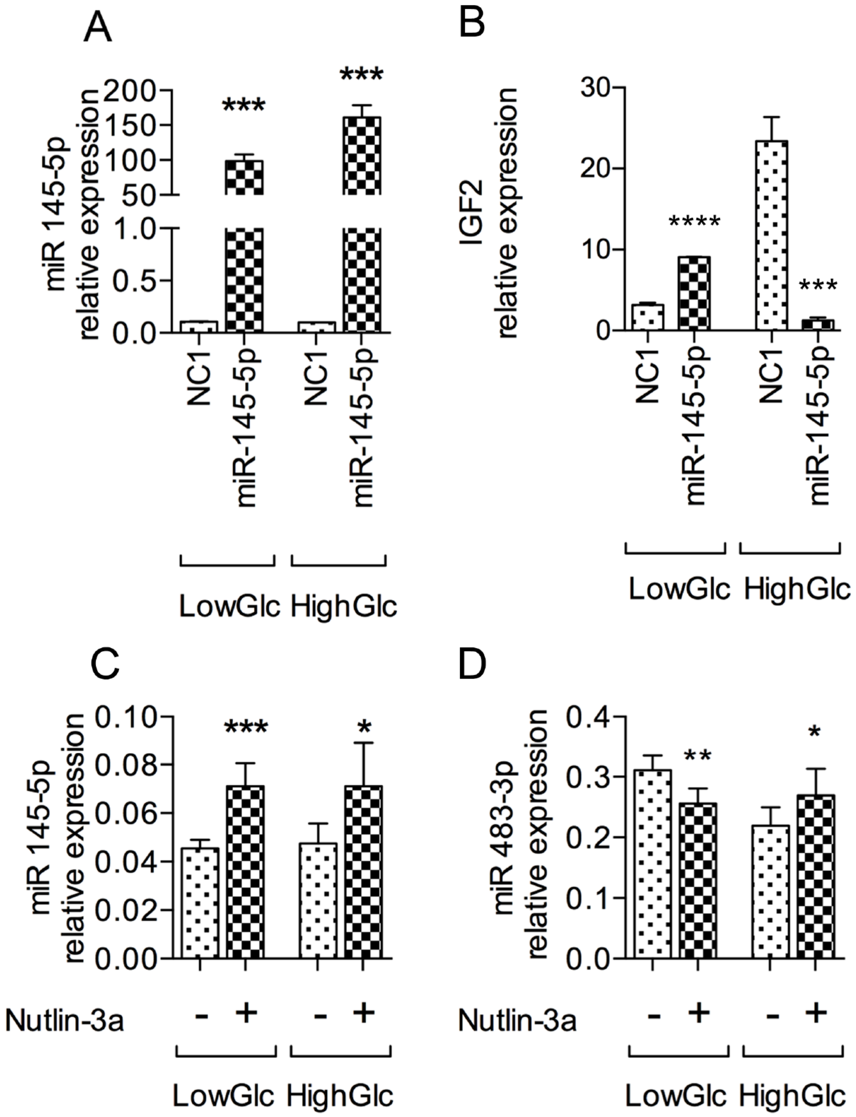

Supplementary Figure S7: A. *miR-145-5p* and **B.** *IGF2* (relative expression by RT-qPCR in HepG2 cells transfected with the scramble sequence NC1 and the mimic *miR-145-5p* in both low (LowGlc) and high glucose (HighGlc) conditions. **C.** *miR-145-5p* and **D.** *miR-483-3p* (relative expressions by RT-qPCR in HepG2 cells treated with 2.5 µM Nutlin-3a for 19 hours in both low (LowGlc) and high glucose (HighGlc).

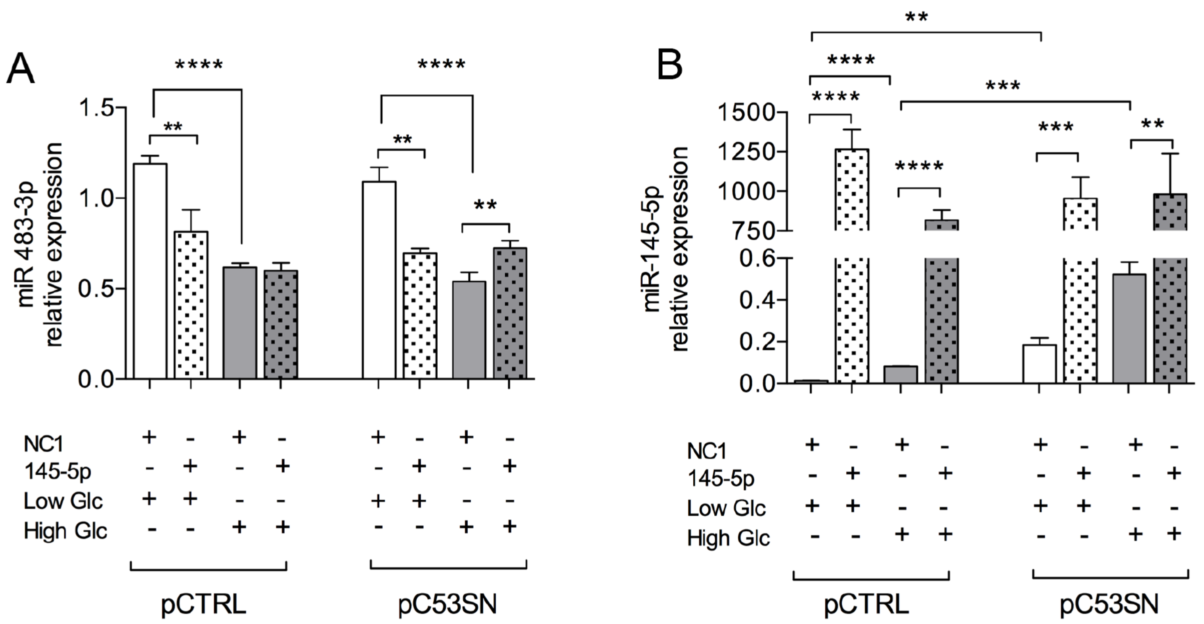

Supplementary Figure S8: A. *miR-483-3p* and **B.** *mir-145-5p* relative expression by RT-qPCR normalized on *RNU44* in Hep3B cells transfected with either *miR-145-5p* or negative control miR (NC1) and either the empty expressing vector (pCTRL) or the TP53 wild type expressing vector (pC53SN); cells were grown in either low (LowGlc) or high (HighGlc) glucose concentration (1 g/L and 4.5 g/L respectively).

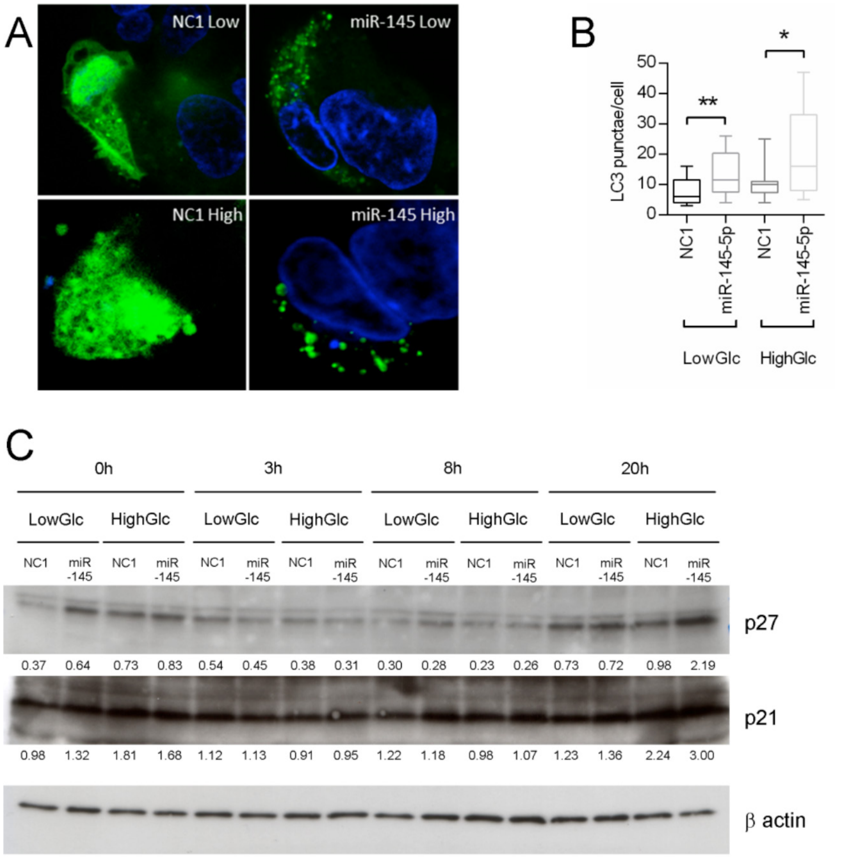

Supplementary Figure S9: A. GFP-LC3 Puncta Formation Assays. To monitoring cellular autophagic activity, HepG2 cells were seeded at midconfluence in an 8 well BD Falcon™ CultureSlides (Becton-Dickinson). The cells were transfected with a GFP-LC3 plasmid and with the scramble sequence NC1, the mimic miR-145-5p or the mimic miR-483-3p, in both low (LowGlc) and high glucose (HighGlc) conditions. After 48h cells were fixed with 2% paraformaldehyde in PBS for 10 min and were washed three times 4 min each with PBS. Following fixation, cells were incubate 60 min in 0,3% Triton™ X-100 (Sigma) in PBS+ (PBS, 5% FBS, 0,02% sodium azide and 10 mg/ml BSA), washed two times with PBS+ (4 min each rinse) and then incubated with DRAQ5 (Cell Signaling Technology) to DNA detection. Coverslip were mounted with SlowFade® Gold antifade reagent (Life Technologies). All confocal images were obtained using a Carl Zeiss LSM510 META system equipped with a Zeiss Axiovert 200 upright microscope and a Plan Neofluar oil-immersion objective (63X). **B.** Quantification of GFP-LC3 puncta per cell was performed using Image J software in more than 20 cells for each condition. *P* value is the result of the unpaired t test two-tailed. Error bars represent SD. **C.** Western blot analysis of HepG2 cells transfected with miR-145-5p mimic (miR-145) or negative control (NC1) in both glucose conditions (LowGlc and HighGlc) to determine the protein expression levels of p27 and p21 at 0, 3, 8 and 20 hours after transfection.

Supplementary Table S1: Characteristics of HCC patients enrolled in the study

**CL**	40
**HCC**	21
**NL**	1

**Total patients (HCC)**	21
**Males**	12
**Females**	5
**Unknown**	4
**HBV +**	4
**HBV -**	14
**Not determined**	3
**HCV +**	13
**HCV -**	6
**Not determined**	2
**Ethanol abuse +**	3
**Ethanol abuse -**	14
**Not determined**	4
**HCC grade 1**	0
**HCC grade 2**	2
**HCC grade 3**	12
**HCC grade 4**	0
**Not determined**	7
**HCC size<50 mm**	12
**HCC size >=50; <100 mm**	3
**HCC size >=100 mm**	1
**Not determined**	5

Abbreviation: HBV, hepatitis B virus;
HCV, hepatitis C virus;
ethanol, history of ethanol abuse;
Grading of the HCC was assessed according to Edmonson and Steiner's criteria.
